# The Potential of Automated Assessment of Cognitive Function Using Non-Neuroimaging Data: A Systematic Review

**DOI:** 10.3390/jcm13237068

**Published:** 2024-11-22

**Authors:** Eyitomilayo Yemisi Babatope, Alejandro Álvaro Ramírez-Acosta, José Alberto Avila-Funes, Mireya García-Vázquez

**Affiliations:** 1 Instituto Politécnico Nacional, Centro de Investigación y Desarrollo de Tecnología Digital, Tijuana 22435, Mexico; 2 MIRAL R&D&I Multimedia, San Diego, CA 92154, USA; ramacos10@gmail.com; 3 Instituto Nacional de Ciencias Médicas y Nutrición Salvador Zubirán—INCMNSZ, México City 14080, Mexico; alberto.avilaf@incmnsz.mx

**Keywords:** artificial intelligence, automated cognitive assessment, cognitive assessment, cognitive impairment, digital cognitive assessment, technology

## Abstract

**Background/Objectives:** The growing incidence of cognitive impairment among older adults has a significant impact on individuals, family members, caregivers, and society. Current conventional cognitive assessment tools are faced with some limitations. Recent evidence suggests that automating cognitive assessment holds promise, potentially resulting in earlier diagnosis, timely intervention, improved patient outcomes, and higher chances of response to treatment. Despite the advantages of automated assessment and technological advancements, automated cognitive assessment has yet to gain widespread use, especially in low and lower middle-income countries. This review highlights the potential of automated cognitive assessment tools and presents an overview of existing tools. **Methods:** This review includes 87 studies carried out with non-neuroimaging data alongside their performance metrics. **Results:** The identified articles automated the cognitive assessment process and were grouped into five categories either based on the tools’ design or the data analysis approach. These categories include game-based, digital versions of conventional tools, original computerized tests and batteries, virtual reality/wearable sensors/smart home technologies, and artificial intelligence-based (AI-based) tools. These categories are further explained, and evaluation of their strengths and limitations is discussed to strengthen their adoption in clinical practice. **Conclusions:** The comparative metrics of both conventional and automated approaches of assessment suggest that the automated approach is a strong alternative to the conventional approach. Additionally, the results of the review show that the use of automated assessment tools is more prominent in countries ranked as high-income and upper middle-income countries. This trend merits further social and economic studies to understand the impact of this global reality.

## 1. Introduction

Over the years, there has been an increase in the use of cognitive screening tools, particularly among older adults, due to the need to provide better management for individuals with impaired cognition. Cognitive impairment is a major symptom in neurodegenerative diseases and can vary in severity, from mild to severe as seen in dementia cases [1]. Consequently, cognitive screening in primary care centers [2] plays a crucial role in the early detection of cognitive impairment [3,4], thereby enhancing early intervention, management, and patient outcome [4]. Common causes of cognitive impairment include neurodegenerative diseases such as Alzheimer’s disease (AD), Parkinson’s disease, vascular dementia, and brain injury, among others [1,5]. Treatment for cognitive impairment depends on its cause, and currently, no curative pharmacological treatments are available [5].

In clinical settings, cognitive assessment is conducted through a combination of interviews, standardized cognitive tests using screening tools like the Mini-Mental State Examination (MMSE), observational assessment, laboratory, and imaging tests [6]. The current cognitive screening tools, predominantly pen-paper-based, must be administered by highly skilled professionals or specialists and are not without challenges. These challenges underscore the need to automate cognitive assessment to improve efficiency and accessibility. With technological advancements [7] and increased effectiveness observed in health care, automating cognitive screening is a promising solution. This can assist and support healthcare professionals in various tasks with NO intention of replacing them [8].

Many studies have explored the use of technology, as seen in technological designs such as games [9,10], technological devices such as computerized test batteries [11,12,13], and wearable technologies [14], in assessing cognitive function with improved precision and scalability. Some others have applied artificial intelligence algorithms in medical data analysis to predict cognitive decline [15,16]. Previous reviews have analyzed the application of AI and machine learning in the analysis of dementia-related data [17], standardization and automation of testing [18], and predictive models for Alzheimer’s disease (AD) risk using public medical databases like ADNI [19]. Some other studies explored the state of computer-based cognitive testing [20,21] and digital cognitive assessment [22,23]. Additionally, some studies compared computerized tests and pen-paper-based tests in detecting MCI and dementia [24] and the primary care physicians’ view on computer-based assessment [25]. This review article highlights the potential of automated cognitive assessment by presenting an overview of existing tools, explaining the diverse mobile and digital applications of technology and AI, ranging from digital neuropsychological test batteries to technology-based tests, wearable and nonwearable devices, and computer and smartphone applications. In addition, we present a comparative analysis of both conventional and automated assessment approaches and briefly discuss the strengths (by stating the performance metrics) and limitations of both, with an emphasis on the potential contributions of the automated-driven cognitive assessment tools for healthcare providers, patients, caregivers, and society at large. This is to build trust in the use of these automated approaches among healthcare professionals.

The rest of the paper is organized as follows: Section 2 details the process of article selection, search strategy, inclusion and exclusion criteria used in our review. In addition, we detailed some background information on some terms and the common conventional assessment tools. Section 3 details our analysis of existing automated tools and the five categories into which we grouped these automated tools. In this section, we further compare conventional and automated tools and discuss their advantages. Section 4 provides the discussion and analysis of our findings, the limitations of the review, and our opinion of the tools. We draw some conclusions based on our analysis in Section 5.

## 2. Methods

**Figure 1:**
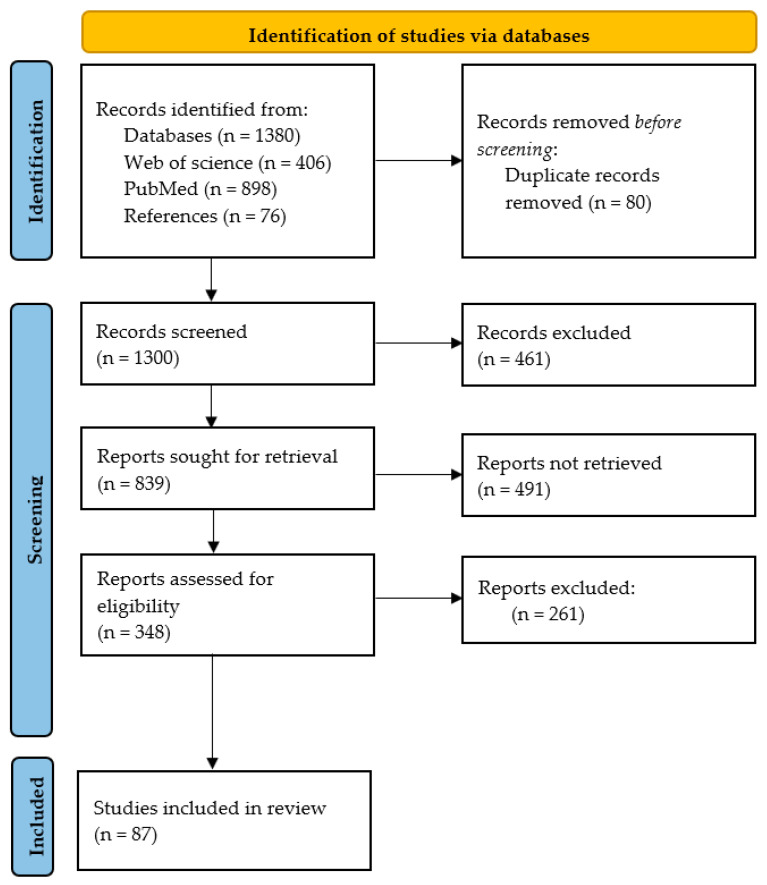
PRISMA flow chart adapted for this study.

This systematic review was conducted without being registered in a public registry. The protocol was developed using PRISMA (preferred reporting items for systematic reviews and meta-analyses) guidelines, as shown in Figure 1. Several articles found in public and academic repositories were put together. Articles discussing automated cognitive assessment in all ranges of diseases were considered. In addition to this, we searched for references to selected articles and performed a manual search for additional papers.

### 2.1. Search Strategy

Two central data repositories, Web of Science and PubMed, which cover biomedical, healthcare, and interdisciplinary research and have a well-rounded collection of high-quality and high-impact, peer-reviewed articles, were searched for studies of interest using a broad range of keywords. The keywords used in our search include: “Computerized trail making test”, “Electronic trail making test”, “comparison of screening tools”, “Cognitive impairment”, “automated assessment”, “Automated assessment of cognition”, “Automated assessment of cognitive impairment”, “Automated assessment of cognitive dysfunction”, “Computerized assessment of cognition”, “Computerized assessment of cognitive impairment”, “Computerized assessment of cognitive dysfunction”, “Automatic cognitive screening”, “Convolutional neural networks to predict cognitive impairment”, “Convolutional neural networks to predict cognitive dysfunction”, “Convolutional neural networks to predict cognition”, “Deep learning to predict cognitive impairment”, “Deep learning to predict cognitive dysfunction”, “Deep learning to predict cognition”, “Machine learning to predict cognitive impairment”, “Machine learning to predict cognitive dysfunction”, “Machine learning to predict cognition”, “Artificial intelligence to predict cognition”, “Artificial intelligence to predict cognitive impairment”, “Artificial intelligence to predict cognitive dysfunction”, “Digital cognitive assessment”, and “Computerized cognitive testing”. Relevant studies were retrieved and selected for this review based on these keywords and date ranges (January 2000–June 2024). Only articles published in the English language were considered. The retrieved articles were screened based on the titles, abstracts, and availability of full text of articles, and duplicates were removed. The references of the included articles were consulted in some cases to strengthen and support the objective of this article. As shown in Figure 1, the final number of articles included in this study is 87 research papers.

### 2.2. Exclusion and Inclusion Criteria

A systematic review was used in selecting studies that fit into the search criteria, and articles that met any of the following criteria were excluded from this review:

1. Studies conducted in a language other than English.
2. Studies that have focused on automated cognitive assessment using medical imaging data, such as magnetic resonance imaging (MRI), positron emission tomography (PET), and computed tomography (CT) scans.
3. Studies assessing cognitive impairment associated with diseases, such as HIV, cancer, stroke, peri- and post-operative procedures, etc.
4. Studies of cognitive assessment in children, adolescents, or nonhuman participants (for example, monkeys and chimpanzees).
5. Articles whose full text was not freely available online.
6. Studies that discuss detection or diagnosis within the scope of conversion from MCI to AD.
7. Studies that provide a limited description of data modalities, subjects, AI techniques, devices, or performance metrics.

For inclusion in this review, studies that met the following criteria were added to this study and reviewed.

1. Studies assessing the diagnosis of cognitive impairment or cognitive function associated with neurodegenerative diseases.
2. Studies distinguishing between control and cognitively impaired participants.
3. Studies predicting cognitive scores with artificial intelligence algorithms or statistical analysis using non-neuroimaging data.
4. Studies comparing the conventional approach of assessment with automated assessment.
5. Studies that discuss digital, computerized, or automated assessment of cognitive decline.

After considering the inclusion and exclusion criteria, 87 articles were considered for this study. These 87 research articles were carried out in 27 countries with a study population covering diverse categories of over 50,000 study participants. The study participants include 7765 participants with Alzheimer’s and dementia, five with dementia with Lewy body, 289 with frontotemporal dementia, 19,992 with mild cognitive impairment, 115 with Parkinson’s disease, three with Parkinson’s disease with MCI, 41 with cognitive frailty, five experiencing cognitive difficulty, six at risk of cognitive difficulty, 80 at risk of cognitive difficulty, 15 with a functional memory disorder, 9876 with functionally impaired, 13,443 with control/normal, 145 with schizophrenia, 300 with systemic lupus erythematosus, 454 with ischemic, 100 with multiple sclerosis, 25 with multiple system atrophy with predominant cerebellar ataxia, eight with multiple system atrophy with predominant parkinsonism, and 10 with other neurological disorder.

### 2.3. Definitions of What Is Known

#### 2.3.1. Background and Concepts

Depending on the clinical stage of the disease, disability in instrumental activities of daily living (IADLs) is common across all syndromes of dementia [26]. These IADLs are tasks necessary to live independently and require a higher level of cognition. A decline in the ability to perform IADLs is a significant marker of cognitive decline [21]. For cognitive assessment, a comprehensive evaluation is carried out by collecting information from physical, neurological, and mental status examinations to understand better the extent of the deficit experienced by the individual [5]. Healthcare professionals review the patient’s clinical history and administer cognitive screening tools, with some common tools discussed in Table 1. A challenge with these tools is their inability to identify subtle changes [27]. Additional information is often sought from family members or caregivers regarding an individual’s cognitive abilities and changes in behavior.

With advancements in technology, like smart and portable devices, wearable sensors, robust software, and artificial intelligence algorithms, automation continues to gain access to all fields [28], including healthcare [29]. Automation involves the use of a system or device to partially or fully accomplish the same function manually done either partially or fully by humans [30]. Automating the manual evaluation of cognitive impairment is likely beneficial to healthcare professionals and individuals as automation promotes efficiency and increases accuracy. Over the years, various automated cognitive assessment approaches have been developed [31,32,33,34]. Several authors have examined and assessed the performance of the pen-paper-based approach, along with technology-based or digital devices, for the assessments of cognitive impairment [35,36]. However, these approaches have not been widely adopted in clinical settings, especially in low- and middle-income countries, where the conventional pen-paper-based method remains dominant. This review analyzes existing automated cognitive assessment methods reporting their performance metrics. Overall, the automated assessment tools aim to reduce human error, streamline evaluation, improve access to timely assessment, and support clinicians in decision-making.

#### 2.3.2. Conventional Assessment Tools

Several conventional tools exist for evaluating cognitive and functional impairment. Cognitive assessments using standardized tools are part of a comprehensive evaluation to guide diagnosis, treatment planning, and intervention strategies. As mentioned earlier, healthcare professionals, including neurologists, geriatricians, psychologists, and occupational therapists, often administer many of these tools to assess cognitive and functional impairment in clinical settings. Some of the commonly used conventional assessment tools are shown in Table 1, and a summary of the domains tested by each tool is mentioned. Indeed, these tools evaluate different cognitive domains such as executive functions, visuospatial ability, and verbal and visual memory [37]. In clinical practice, patients are assigned simple tasks such as naming the current date, identifying everyday objects or pictures of animals, copying a drawing of a shape or objects [38,39], and drawing a clock [38,40]. In the end, each session is scored, and the sum score is calculated and interpreted to ascertain the level of impairment. In addition, details about patients’ performance of simple daily activities are often based on patients, family members, and caregivers’ reports [41]. However, this information may be inaccurate [41], as Loewenstein et al. [42] showed that it is overestimated by caregivers.

**Table 1:** Common conventional assessment tools for evaluating cognitive and functional status.

Tool	Purpose	Domain	Maximum Score Possible	Administration Time
LABIS Graf et al., 2008 [43]	IADL screening (Functional evaluation)	Eight domains: Ability to use telephone, shopping, food preparation, housekeeping, laundry, transportation, responsibility for own medications, and ability to handle finances	8 points	10 to 15 min
Katz ADL Index Katz et al., 1970 [44]	ADL screening (Functional evaluation)	Six domains: bathing, dressing, toileting, transferring, continence, and feeding	6 points	Less than 5 min
MMSE Folstein et al., 1975 [39]	Cognitive screening (Cognitive evaluation)	Five domains: orientation (to time and place), memory (immediate and delayed recall), concentration and attention and calculation, three-word recall, language, and visual construction	30 points	Between 5 to 10 min
Mini-cog Borson et al., 2000 [40]	(Cognitive evaluation)	Two domains: A 3-item recall component and a clock drawing test	5 points	Takes less than 3 min
MoCA Nasreddine et al., 2005 [38]	MCI and Dementia screening	Eight domains: Visuospatial/executive, naming, memory, attention, language, abstraction, delayed recall, and orientation (to time and place)	30 points	Approximately 10 min

ADL—Activity of daily living, LABIS—Lawton and Brody IADL scale, MCI—mild cognitive impairment, Mini-cog (mini-cognitive), MMSE—Mini-Mental State Examination, and MoCA—Montreal cognitive assessment.

Functional assessment measures an individual’s ability to perform specific tasks independently and can be categorized into self-reported and performance-based [45]. Performance-based functional assessments such as timed walks and other tasks related to motor function are an objective alternative to self-reported measures in the form of questionnaires [46]. In this approach, direct observation is required while the patient demonstrates IADL. This approach is difficult to administer in the clinical setting but is suited for academic purposes and yields more accurate results [46]. Self-reported measures are primarily used [45]. Functional assessment is done using standardized tools like Lawton’s IADL scale [43] and KATZ ADL [44], while cognitive assessment tools include MoCA [39] and MMSE [39], among others.

These conventional tools face several limitations, including the time required to score the patient and the need for a specialist to administer the test [47,48,49]. They are unsuited for long-term tracking due to the lack of alternative forms [50]. They cannot be modified to an individual’s competence level and are unsuitable for retesting due to the static nature of the questions [49]. Other challenges are associated with humans, some of which are biased, as observed in caregivers’ reports of impaired AD patient’s functional ability, fatigue, and distraction during the assessment [42]. These challenges pose difficulties in accurately diagnosing patients. Moreover, premorbid status, such as intelligence or education, dramatically affects the validity of some tools like MMSE [4].

## 3. Results

### 3.1. Automated Assessment Tools

The advent of technology has made it easier to assess cognitive domains. Existing automated approaches for assessing cognitive function include digital versions of established standardized tests and new computerized tests. These automated approaches leverage technological approaches to enhance cognitive assessment. These tools often provide objective and quantitative measures, allowing efficient and standardized evaluations. While several tools assess various cognitive domains, a common focus among them is the assessment of the state of memory and function. In many of these studies, automated cognitive assessment is conducted using various platforms. We carefully considered the existing tools and categorized them into five based on the design and approach of analysis. They are game-based, digital versions of conventional tools, original computerized tests and batteries, virtual reality/wearable sensors/smart home, and artificial intelligence-based (AI-based) tools. Each category is further discussed in subsequent subsections. Additionally, a comprehensive table comparing the 87 automated cognitive assessment tools categorized based on this classification is included in Table S1a–e as a reference for readers.

#### 3.1.1. Game-Based

In recent years, several studies have used games beyond the purpose of entertainment, and this has helped in accurately assessing cognitive and functional impairment [9,10,48,51,52]. Here, we discuss using games as a medium for cognitive health assessment. Our analysis is based on 10 articles in which different games assess human cognitive function. This approach is used to assess cognitive and functional skills by assessing correctness, accuracy, and completion of tasks while carrying out some tasks. Some of these tasks include shape matching, visuomotor tracking, and drawing. Devices such as touchscreen computers and tablets are used to administer these tasks, and they are relatively affordable. Lindenmayer et al. [9] used VRFCAT, a game-based environment, to predict functional ability among schizophrenia patients. Significant correlation values of 0.005 and 0.01 with UPSA-B were obtained at baseline and total score, respectively [9]. A serious game was employed among AD patients to test for cognitive impairment and was found to be user-friendly accommodating the functional deterioration in patients [10]. Some research has also explored the correlation between game-based assessments and the conventional questionnaire approach. An example is Cheng et al. [51], where a game-based system was administered as an automated cognitive assessment tool to 80 participants. The correlation result with the Wechsler Adults Intelligence Scale 4th Edition (WAIS-IV) ranged from 0.34 to 0.51. Some of these tools test judgment ability and memory function and are sensitive to identifying subtle cognitive decline [53]. Although older adults are presumed to be uncomfortable with games due to a lack of game experience. Yang et al. observed that games like MahjongBrain are user-friendly for older adults [54]. This approach offers flexibility with testing and is clinically valuable for assessing cognitive impairment [55]. From our analysis of these articles on the game-based approach of assessment (see Supplementary Table S1), we observe a moderate correlation (greater than or equal to 0.5) of this approach with conventional tools [56] such as MMSE. Some games, like the EVO Monitor, a digital cognitive assessment developed by Akili Interactive Labs (Akili, Boston, MA, USA), are available online and can run on tablets or touchscreen computers [55]. Others, like the NAIHA Neuro Cognitive Test (NNCT) [56] are designed by professional research groups and may be available on request.

#### 3.1.2. Digital Versions of Conventional Tools

Some of the existing conventional tools have been fully digitized, as seen in eMoca [57,58] and MMSE mobile applications [36]. Others digitize parts of existing conventional tools such as the eCDT, mPDT [59], and eTMT [60,61]. This digital format uses an electronic pen/stylus and tablet to perform the same task as the conventional approach, and scoring is based on software or AI models. The automated scoring introduced in the digitized version has greatly improved efficiency and reduced human bias. Here, we analyzed 12 articles that focus on this approach for cognitive assessment. Some digital features measured include pen movement, time of completing the task, and the number of strokes made while performing the task. This digitized version of conventional tools has shown a positive correlation with their conventional counterpart as observed in the MMSE mobile application [36] being r = 0.9, and an adequate convergent validity of 0.84 between the conventional MoCA and eMoCA [57,58]. It is capable of measuring cognition in the same way as the conventional, as observed in the eTMT where the correlation value between the derived scores using the pen-paper TMT and the eTMT range between 0.51 and 0.67 and the intraclass correlation coefficient (ICC) value range between 0.90 and 0.95 [60]. A positive correlation value of 0.651 was observed between pen-paper TMT-B and eTMT-B [62], and the predicted eTMT score correlates with clinical scores at a value of 0.98 [61]. Additionally, it has the potential to screen MCI based on its performance, as seen in eCDT [35], as it demonstrates a higher performance (sensitivity) compared to the conventional CDT, with a difference of 0.18 in sensitivity value [63]. Cognitive assessments using the digitized version can be conducted using mobile devices, tablets, and computers. Though older adults have limited digital skills, this method is promising, as it offers wider accessibility to cognitive evaluations. In addition, it provides ease of adaptation to different languages [36]. Furthermore, it supports group screening wherein physicians can administer screening tools due to the portability of the software used [64]. Free versions of digitized tools like the MoCA test are available online for healthcare professionals and academia in multiple languages.

#### 3.1.3. Original Computerized Tests and Batteries

The original computerized test and batteries category includes computerized batteries and/or tests. We analyzed 35 articles using these batteries and tests to assess cognitive function. Computerized batteries are a collection of standardized cognitive tests that are fully automated and assess several cognitive domains. They are administered through a computer. These batteries are not adapted from traditional/conventional existing tools. They have shown a moderate correlation with the conventional approach, as in the case of Minnemera, which is within the range of 0.34 and 0.67 [27]. ANAM was found to be more effective than MMSE in detecting cognitive impairment among heart failure patients [65]. Computerized cognitive tests are designed to assess cognitive and/or executive function using software or applications to generate scores and sometimes interpret results on a computer. Some of these tools have shown high performance based on high correlation with standardized tools and their sensitivity and/or specificity. CST (computer self-test) [66], performed better than MMSE and mini-cog in classifying cognitively impaired subjects, achieving 96% accuracy, while MMSE and mini-cog had values of 71% and 69% respectively. Computerized cognitive screening (CCS), [67] showed a high correlation with conventional MoCA with a value of 0.78 and a sensitivity value of 0.94, similar to MoCA’s value of 0.95 while screening for cognitive impairment. In addition, mSTS-MCI [68] also showed a high correlation value of 0.773 with the Korean version of MoCA and a higher sensitivity and specificity in screening MCI. Like other technological tools, older adults may be unfamiliar with and may not be interested in using these tools for assessment. However, computerized batteries are good tools for cognitive assessment since they have standardized administration and are sensitive to subtle change. Some computer-designed tests, like the Hong Kong-vigilance and memory test (HK-VMT) [69], are available online and can be used on touchscreen computers.

#### 3.1.4. Virtual Reality/Wearable Sensors/Smart Home Technologies

This category refers to the virtual reality approach and smart home technologies. We analyzed 10 different articles in this category. Virtual reality has also been used to simulate real-life tasks and assess patients based on their performance on these simulated tasks. Smart home technologies with sensors to capture the needed information have been used to gather information related to everyday life. These smart home technologies and virtual reality systems extract features, analyze them, and make assessments by considering features such as the time taken to carry out an activity and the completeness of the activity, to mention a few. Analysis/prediction based on the information gathered uses statistical or artificial intelligence algorithms. Studies have shown that this approach can potentially predict patient cognitive health [70,71]. CAAB (Clinical Assessment using Activity Behavior) showed a high correlation value of 0.72 with the cognitive scores provided by the clinician [70,71]. CAVIRE (Cognitive Assessment by Virtual Reality), a virtual reality system, takes less time to complete the assessment than the conventional pen-paper-based MoCA, with a mean difference of 74.94 s in assessing healthy Asian adults [14]. The high cost of virtual reality software, sensors, and technological equipment associated with this approach is a significant drawback. Nevertheless, this approach provides real-world or at-home data collection and monitoring opportunities. The ability to track daily activities can support identifying changes in cognitive function.

#### 3.1.5. Artificial Intelligence-Based (AI-Based) Tools

Artificial intelligence has emerged as a promising tool in healthcare, especially for analyzing medical data in cognitive assessment [72]. It has been used to screen, predict, and analyze large datasets of cognitive test results, digital biomarkers, and medical records. Artificial intelligence (AI)—based techniques for cognitive assessment have been employed in several ways, from scoring [73] to analyzing [74] and predicting [75] cognitive impairment. This approach is often applied to different types of data, such as imaging, behavioral, and non-neuroimaging data. This category focuses on AI-based approaches using non-neuroimaging data, and we analyzed 24 articles. An additional eight articles were already categorized into one of the four classes above; however, since the authors used AI for data analysis, we have also included them in this category. Different AI algorithms have been applied to different data by different authors. Some authors applied machine learning algorithms to speech data [15,76,77,78,79,80] for analysis and prediction. Others applied deep learning algorithms to image data and assessed patients by automatically scoring drawn images [74,81,82,83]. These algorithms learn from datasets, extract the necessary features and make predictions. Some of these techniques used in cognitive assessment include machine learning [34], deep learning [82], and natural language processing [80], among others. Machine learning techniques involve the use of Bayesian methods, support vector machines, random forests, logistic regression, and decision trees, among others [34,48,84]. Deep learning algorithms use deep neural networks and require large data for training [82]. The natural language processing technique is used to understand human verbal and written communication [80]. This approach is used to analyze audio or speech recordings [79,80]. It includes speech recognition and sentiment analysis. Sato et al. [74] built a CDT-based deep neural network (DNN) model using machine learning for scoring drawn CDT, and a high-performance metric of approximately 90% was achieved for executive dysfunction and 77% for probable dementia. Using convolutional neural network algorithms, Youn et al. [75] achieved a 71% accuracy for classifying control, mildly and severely impaired persons CDT and RCFT-copy data. Nakaoku et al. [85] developed a predictive model using power monitoring data to detect cognitive impairment and achieved good performance values of 0.82, 0.48, and 0.96 for accuracy, sensitivity, and specificity, respectively. Rykov et al. [81,86] developed an explainable self-attention deep neural network which achieved an accuracy of 0.81. A deep learning algorithm was applied to CDT, and an accuracy of 0.97 and 0.99 was achieved for screening and scoring, respectively [82]. These tools have shown potential in analyzing cognitive performance data to provide predictions for supporting diagnosis. In addition, the models are often available online but will require fine-tuning for use. The major drawback is the need for a large volume of quality labeled data for training AI models. Furthermore, there is a high need for the predictions made by AI models to be interpretable, but with the help of explainable AI [81], this challenge can be overcome. AI-based category offers improved efficiency, improved scoring accuracy, prompt assessment, and overall, early detection.

In all five categories, automated cognitive assessment has proven to be a strong alternative based on the comparative analysis report in the subsequent subsection.

### 3.2. Comparative Analysis of Automated and Conventional Cognitive Assessment Tools

Here, we evaluate several studies detailing the different cognitive screening tools (Table 2 and others in the Supplementary Materials). Table 2, an excerpt from the Supplementary Materials, presents an analysis of the tools based on the performance metrics reported by the authors. The first part of Table 2 compares conventional and automated approaches together. Here, the comparison was based on performance metrics reported by the authors. These performance metrics include sensitivity, specificity, and, where available, AUC. The latter part presents individual automated tools and their performance metrics. We report the success of these screening tools based on the performance metrics (correlation (r), area under the ROC curve (AUC), sensitivity (sens), and specificity (spec)) provided by the author. Additional metrics like accuracy, precision, and other statistical measures are reported as described by the author in the Supplementary Materials.

As MoCA is considered a better screening tool for MCI than MMSE in the literature [87], we selected from Table S1a–e more works comparing the performance of the automated approach with MoCA and presented this in the summarized Table 2 below. This table shows the performance of both automated and conventional approaches. According to the literature [88], sensitivity is the ability of a screening tool to detect true positives, that is, people with a condition of interest which in this case is cognitively impaired. At the same time, specificity is the ability of a screening tool to detect true negatives, that is, identifying people who do not have the condition of interest, in this case, those who are not cognitively impaired. A high sensitivity (sens) indicates a high probability or effectiveness in identifying cognitively impaired individuals (true positive). On the other hand, a high specificity (spec) indicates a high probability or effectiveness in identifying individuals who are not cognitively impaired (true negative). Correlation measures the association or relationship between two variables [89]. The correlation (r) in this context indicates the degree to which the conventional and automated approaches relate/agree. The area under the ROC curve value (AUC) measures the probability of a model to identify correctly a diseased and nondiseased individual, in this case cognitively impaired and unimpaired individuals [90]. A high area under ROC curve value (AUC) in this context suggests the effectiveness of an approach in distinguishing between different classes or groups of participants. The higher the AUC, the better it is in distinguishing between groups of participants. In Table 2 and the Supplementary Table S1, the correlation, sensitivity, specificity, and area under ROC curve values all range from 0 to 1. Values between 0–0.49 indicate low to moderate performance/probability, between 0.5 to 0.99 indicate moderate to high performance/probability while values of 1 indicate a perfect performance/probability.

**Table 2:** Summarized performance evaluation of cognitive assessment tools.

	Tool	Participant	Domain Assessed By the AA	Comparative Metrics Reported for Both the Conventional Approach (CA) and Automated Approach (AA)		Time Taken to Administer	Observation	Reference
Automated tools compared with conventional tools like MoCA	MoCA (CA) ACE-R (CA) CANS-MCI (AA)	35 participants (20 CN and 15 MCI)	Memory, executive function, and language/spatial fluency	AUC (MoCA) = **0.890** AUC (ACE-R) = 0.822 Sens (CA) = **0.90** Spec (CA)= 0.67 (sens and spec value is for both MoCA and ACE-R)	AUC (CANS-MCI) = 0.867 Sens (AA) = 0.89 Spec (AA)= **0.73**	MoCA ~ 10 min ACE-R ~ 15 min CANS-MCI ~ 30 min	Of the 3 examples cited here, AA and CA appear to have a close and competitive outcome.	[91]
	CDT (CA) CDT (AA)	70 (20 AD, 30 MCI and 20 CN) patients	Executive and visual-spatial function	Sens (CA) = 0.63 Spec (CA) = **0.83**	Sens (AA) = **0.81** Spec (AA) = 0.72	NA		[63]
	MoCA-k (CA) mSTS-MCI (AA)	177 participants (103 CN and 74 MCI)	Memory, attention, and executive function	AUC (CA) = 0.819 Sens (CA) = 0.94 Spec (CA) = 0.60	AUC (AA) = **0.985** Sens (AA) = **0.99** Spec (AA) = **0.93**	mSTS-MCI ~ 10–15 min		[68]
Automated tools with high correlation when compared with the conventional approach	mSTS-MCI	177 participants (103 CN and 74 MCI)	Memory, attention, and executive function. Reaction time is assessed for attention while the other 2 measures performance.	r = 0.773 correlation with MoCA-K (Korean version of MoCA) Sens = **0.99** Spec = **0.93** (sens and spec at optimal cutoff)		10–15 min	Findings reflected in the correlation between both approaches show a positively high association between both.	[68]
	CoCoSc	160 participant (59 CI and 101 CN)	Six subtests covering five cognitive domains including learning and memory, executive functions, orientation, attention and working memory and time- and event-based prospective memory are scored based on completion of the task.	r = 0.71 correlation with MoCA AUC = 0.78 Sens = 0.78 Spec = 0.69		15 min		[92]
	CCS	60 participants (20 CN and 40 mild-moderate dementia but only 34 completed the CCS task)	Three domains were assessed concentration, memory, and visuospatial with related tasks and scored based on correct responses provided in 1 min for each task.	r = 0.78 Correlation with MoCA Sens = 0.94 Spec = 0.60 AUC = 0.94		1 min per task		[67]
	C-ABC (Computerized assessment battery for cognition)	701 participants (422 dementia, 145 MCI, and 574 CN)	Sensorimotor skills, attention, orientation, and immediate memory, among others	r = 0.753 Correlation with MMSE score Sens = 0.77 Spec = 0.71 Average values for distinguishing MCI from CN		~5 min		[33]
	MoCA-CC	176 participants (83 CN and 93 MCI)	Eight cognitive domains: executive function, memory, language, visuoconstructional skills among others	r = 0.93 correlation with MoCA-BJ AUC= 0.97 Sens = 0. 958 Spec = 0.871		~10 min		[64]

AA (automated assessment), AD (Alzheimer’s disease), AUC (area under the ROC curve), CA (conventional assessment or conventional approach), CI (cognitively impaired), CN (cognitively normal/healthy adult), MCI (mild cognitive impairment), NA (not available), r (Pearson correlation), Sens (sensitivity), and Spec (specificity). Please note that the values in bold show the highest value obtained when comparing both the automated and conventional tools based on the performance metrics reported.

In the first three rows, where the performance of both conventional and automated assessment is compared, we observe that of the three studies discussed [63,68,91], both the conventional and automated assessment approaches showed good performance metrics, as detailed above. However, in the first row with MoCA, the conventional approach has a slightly higher sensitivity (0.9) and area under curve value (0.89), while the automated approach has a higher specificity (0.73). This finding of a higher sensitivity with the conventional approach indicates that the conventional approach is highly effective in identifying cognitively impaired persons. On the other hand, the automated approach with higher specificity suggests that it is effective in identifying those who are not cognitively impaired. In the clock drawing test study [68], the automated assessment approach showed higher sensitivity (0.81), while the conventional approach showed higher specificity (0.83). Again, this indicates the effectiveness of the automated approach in identifying cognitively impaired individuals and the conventional approach’s effectiveness in effectively identifying individuals who are not cognitively impaired. In the MoCA study [63], in the third row, the automated approach showed higher AUC (0.985), sensitivity (0.99), and specificity (0.93). This presents the automated approach as a tool capable of effectively identifying cognitively impaired and unimpaired individuals. Overall, in these three studies, the automated approach demonstrated better sensitivity and specificity than the conventional approach.

Considering other automated tools presented in Table 2, like the mSTS-MCI [68], CoCoSc [92], CCS [67], C-ABC [33], and MoCA-CC [64], the result shows high correlation values with the conventional tools with values ranging from 0.71 to 0.93. Also, among these five studies [33,64,67,68,92], the sensitivity values range from 0.77 to 0.99, while specificity values range from 0.61 to 0.93. This result demonstrates that these automated tools can be used as an alternative to the pen-paper approach. The high sensitivity of these tools also shows that they can identify subtle changes, unlike the MMSE, which is known in the literature to have low sensitivity (0.65) [93] compared to MoCA in diagnosing cognitive impairment [87]. The high sensitivity (0.99) [68] achieved with the use of an automated approach, like mSTS-MCI, highlights its potential for supporting early diagnosis leading to earlier intervention.

Based on the performance metrics reported in Table 2, the automated approach of assessment can effectively measure several cognitive domains, like the conventional approach. Further information on more automated assessment tools is provided in Table S1a–e in the annex.

### 3.3. Advantages of Automated Assessment

After reviewing 87 articles that met our exclusion and inclusion criteria, we identified some notable advantages of automated cognitive assessment. Our analysis of these automated screening tools found that there is no need for experts to administer this test as anyone can be trained to operate some of them, like CAVIRE [14], while some others can be self-administered [13,70,94]. Many of these tools can be used remotely at home or in primary healthcare settings and do not require a trained specialist [14], making them more efficient than the conventional pen-paper approach.

They can be standardized and are not affected by human bias [21]. They are more accurate and sensitive tools for screening MCI and are more focused on memory tests [70]. Automated screening and scoring are achievable with these tools using different AI algorithms and software [82]. These tools are scalable [14] and can support triaging individuals, which may relieve health practitioners and promote timely access based on the individual’s severity. Results from this automated assessment can be stored or transferred into patients’ electronic medical record systems [36]. These tools can be used in a very diverse population as language can be switched based on the user’s preference [36]. This can be a form of great support for clinicians [36]. These tools can support increased access to cognitive assessment [58,61].

Additional features like the reaction time can be captured, further supporting other research focused on behavioral analysis [95]. Some additional digital/performance features related to mobility and time, such as the quality of tasks performed and time taken to transition the stylus, among others, may help monitor other cognitive processes not captured by paper [61]. Due to this automatized screening and scoring, they are practical for assessing large cohorts [96]. These tools can potentially increase the reliability and efficiency of cognitive assessment [53]. Some of these tools possess high sensitivity and specificity and are highly efficient in correctly discriminating between MCI and CN, as seen in CAMCI [13] and mSTS-MCI [68].

Overall, this automated cognitive assessment approach is efficient and cost-effective, supports standardization, and prompt assessment, increases access to assessment, encourages frequent testing, and can be self-administered. The feature of automatized scoring and screening makes it suitable for screening large cohorts, eliminating human bias, and hence, reliable and efficient. All of these give the automated cognitive assessment an edge over the conventional pen-paper approach.

## 4. Discussion

In this review, we evaluate the potential of automated cognitive assessment based on the performance metrics reported alongside the advantages presented with the use of diverse automated cognitive assessment tools. These two (the performance metrics and the advantages) present it as a strong alternative to conventional tools. The effectiveness of the reviewed automated assessment tools in screening for cognitive impairment is demonstrated in the high sensitivity, specificity, accuracy, and area under curve value. From our analysis of the 87 articles, we categorize the automated cognitive assessment tools into five groups: game-based, digital versions of existing cognitive tools, computerized tools and batteries, virtual reality/wearable sensors/smart home technology, and artificial intelligence-based tools. As shown in Table S1a, six (6) of the reviewed articles belong to the game-based method. Of these methods, Panoramix [48] shows a promising performance of 100% in identifying cognitive impairment. In addition, Evomonitor [55] showed a moderate correlation with a brief assessment tool (SDMT), while NAIHA [56] showed a moderate correlation with MMSE. Furthermore, twelve (12) of the total reviewed articles were categorized as digitized versions of existing conventional tools with significant performance, such as high correlation with conventional tools, as observed in MMSE (app) [36] and MoCA-BJ [64]. In addition, high sensitivity and specificity (>85%) are seen in MoCA-BJ [64], ePDT [59], and eCDT [35]. The computerized tests and battery method account for the largest category, totaling thirty-five (35). Of these methods, high correlation with conventional tools (with value > 0.7) was observed in [33,67,68,73,92,97,98,99,100,101], high sensitivity and specificity (with a value > 60%) in [11,33,66,69,91,102,103,104,105,106], showing the capacity to correctly identify impairment, high area under curve value (>0.7) in [66,67,68,69,91,92,102,103,105,107,108,109,110] and others showing moderate performance and greater than 80% correct classification in [12]. The virtual reality/wearable sensors/smart technologies have ten (10) articles of the total reviewed articles, of which high sensitivity and specificity (value >80%) were observed in [13] and a moderate correlation (>0.5) between predicted and observed/clinician scores in [70,111]. The last category, the artificial intelligence-based method has twenty-four (24) articles out of the total articles reviewed with potentially high accuracy (>70) as seen in [34,74,75,76,81,82,83,85,112,113,114,115,116], AUC value (≥ 70) as seen in [76,77,78,84,114,115,117,118], moderate correlation with conventional tool [86,119] and relatively high sensitivity, specificity, and accuracy as seen in [76,114,115,116,120].

The efficacy of these tools is evident in the high sensitivity and specificity recorded in tools such as CANTAB, CAMCI, ANAM, Cogno-Speak, and BrainCheck [11,13,35,59,63,102,104,120], and they are as reliable as conventional tools in screening cognitive impairment as in the case of eMoCA [57]. This further underscores the reliability of automated assessment in accurately screening for cognitive impairment. In addition, automated tools like CANS-MCI, CST, HK-VMT, and BHA [66,69,91,103,105,114] displayed high sensitivity, specificity, and the area under the curve, showcasing it as a powerful tool for correctly identifying and classifying individuals with or without cognitive impairment. Other tools like the CogEvo and Brain on Track [69,78,107,108,109,121] can also classify individuals with or without cognitive impairment based on the high AUC values reported. Moreover, some of these automated tools, eMoCa, CoCoSc, mSTS-MCI, and CCS, have shown a strong correlation with conventional tools like the MoCA [36,57,60,61,67,68,73,92], indicating that they similarly measure cognitive function, thereby suggesting it as a possible alternative.

Of the 27 countries identified in the 87 papers reviewed here, 71% belong to high-income countries, and the remaining 29% are categorized as upper middle-income countries according to the World Bank country classifications by income level for 2024–2025. The low-income and lower middle-income countries have yet to adopt the automated approach, which may be due to a lack of basic infrastructure such as uninterrupted electricity supply and poor/average access to the internet. It is projected that by 2050, 68% of the global prevalence and burden of dementia will take place in low and middle-income countries [122]. Adopting this automated assessment approach and encouraging its use by clinicians may support early diagnosis in low, lower middle, and high-income countries.

Furthermore, our analysis of 87 papers and their metrics shows that 22 automated tools have documented correlation values with conventional tools and four with significant correlation whose value was not reported. This positive correlation between conventional and automated tools shows that both tools are consistent and related in their measurement of cognitive impairment. Additionally, three of the total articles report their ability to distinguish between cognitively impaired and unimpaired with no value to support this. Nineteen of the total reviewed papers were proven to be potentially useful for cognitive screening. One has a sensitivity and specificity greater than 60%, and 20 papers have a sensitivity and specificity greater than 70%. These high values of sensitivity and specificity indicate that the tools are capable of correctly identifying cognitive impairment and identifying unimpaired, respectively. Ten have accuracy values greater than 70%, one records high sensitivity and specificity without specific values, seven record AUC values greater than 70%, while two records over 80% correct classification of MCI and control group. This shows that the automated approach is a reliable alternative to the conventional approach, and considering its advantage of increased access to tests, its use should be greatly encouraged, especially in primary healthcare centers where specialists may not be readily available.

Compared with the pen-paper-based test, automated assessment offers cost-effectiveness, the ability to store patients’ data, and accurate recording of responses [21], is consistent with other reviews. Evaluating cognitive function is crucial for diseases associated with memory loss, as this information is essential for decision-making. Automating the assessment of cognitive status may facilitate the prediction of cognitive impairment, which could help in the diagnosis of neurodegenerative diseases such as Alzheimer’s. These automated tools like eMoCA, CCS, and CANS-MCI, among others, can be used for monitoring cognitive health [71], screening for probable dementia or decreased executive function [74], and scoring and screening of cognitive impairments [82]. Additionally, most of these automated approaches are appropriate for natural (smart home technologies) [71] and clinical environments. Some automated approaches are more effective than the conventional method, like MMSE, for screening cognitive impairments, as observed using ANAM [65]. With automated scoring, results can be described in a way that is easy to understand and interpret [123]. This approach offers an opportunity to measure subtle changes in executive functions [63], subtle changes or alterations in language features that may not be detectable by conventional methods [15], thereby increasing early detection of cognitive impairment.

Although prior experience or familiarity with technology and related devices is observed to aid performance, as observed in [32], where individuals with more experience using touchscreen devices performed better on eMoCa compared to their contemporaries without the experience. On the other hand, Scalon et al. [67] found no difference in automated assessment scores between those with and those without prior computer experience. This issue is likely not to be a problem in the future, as the current generation is increasingly familiar with the use of technology and digital devices. Automating the assessment of cognitive function shows potential that gives room for its inclusion in the diagnosis and detection of cognitive decline.

### 4.1. Limitations

One limitation of this review lies in the potential for publication bias, as the inclusion of articles was restricted to those available in the selected databases with free full-text and within the specified timeframe (January 2000–June 2024). The exclusion of studies documented in other languages different from English may have led to the removal of the potential contribution of such works. Additionally, the time it takes to complete these automated tests and the cost associated with these approaches were not stated in this review due to the limited availability of this information. It is fair to mention that most of these automated approaches cannot be used as a standalone diagnostic tool but as support for clinical decision-making.

### 4.2. Authors’ Opinion

The implication of adopting technology-driven cognitive assessment tools is discussed in this section. Some of these automated assessment approaches, such as eMOCA [57], eCDT [35,63], eTMT [60,61], ePDT [59], CoCoSc [92], and CCS [67], among others, may be challenging to people with visual impairment. This automated approach may also pose a challenge to individuals who are not familiar with computers or technology in general. However, as the current generation ages, technology will likely become less of a challenge as people, even in developing or underdeveloped countries, interact with technology daily. These tools only support, not replace, clinical assessment tools or healthcare practitioners. With the future in view, clinicians and individuals should embrace this evolving approach to encourage technology developers, improve the performance of automated tools, and overall improve access to care and treatment. Continuous collaboration between medical and technology experts will further strengthen the potential of these automated tools and facilitate their acceptance.

## 5. Conclusions

The potential of the automated tools identified in this review is evident in their ability to accurately classify individuals with or without cognitive impairment and their correlation with existing conventional tools, as shown by the 87 articles. These tools are scalable and readily available, thereby increasing accessibility for screening with minimal or no human intervention, as many of these tools can be self-administered. This capability can lead to early diagnosis and intervention, ultimately improving individuals’ quality of life. Some studies identified longer test completion time [58] and limited familiarity with the devices or technological approaches used as disadvantages, which may impact performance or misrepresent the patient’s cognitive status [32]. However, the advantages of the automated assessment were evident throughout this review and outweighed the identified weaknesses or disadvantages. These automated approaches have shown significant potential for early screening before other tests are conducted, facilitating early intervention and allowing for comprehensive patient care plans. The automated approaches reviewed have shown comparable diagnostic performance to their pen-paper-based counterparts in all the articles included in this study, with correlation values ranging between 0.4 to 0.9 and sensitivity and specificity ranging between 0.7 to 0.9. Collectively, these studies highlight the promising impact of automating the assessment of cognitive function. Considering the performance metrics reported (sensitivity, specificity, accuracy, correlation, and area under curve), these tools offer timely interventions, improved access to care, prompt triaging, and effective patient monitoring, which can be highly beneficial for clinical trials. High correlation, high sensitivity, and specificity values support the validity of these automated tools and show their potential use for cognitive assessment.

Integrating technology into healthcare practice, especially for diagnosing cognitive impairment and analyzing medical data for predicting cognitive impairment, can transform the process, enhance diagnosis and treatment, and improve patient outcomes. Collaboration between healthcare professionals and technology developers will further strengthen the use of technological tools and algorithms and address the challenges associated with their use. Achieving this will greatly advance the diagnosis of diseases related to cognitive and functional impairment. These automated approaches offer the possibility of developing clinical devices that are highly sensitive, noninvasive, and cost-effective for testing cognitive decline. This review shows that automated assessment tools are more useful in high- and middle-income countries. Future work may consider evaluating the reasons for the low utility of automated assessment in low- and lower income countries.

## Supplementary Materials

The following supporting information can be downloaded at: https://www.mdpi.com/article/10.3390/jcm13237068/s1, Table S1: Performance evaluation of automated cognitive assessment tools based on the 5 categories; (a) Game-based, (b) Digitized version of some conventional tools, (c) Original computerized tests and batteries, (d) Virtual reality/Wearable sensors/Smart home technologies, and (e) Artificial intelligence-based (AI-based) techniques, references [124,125,126,127,128,129,130,131,132,133,134,135,136,137,138,139,140] are cited in Supplementary Materials.

## Abbreviations

The following abbreviations are used in this manuscript:

AA	Automated Assessment
Accu	Accuracy
ACE-R	Addenbrooke’s Cognitive Examination-Revised
AD	Alzheimer’s Disease
ADL	Activity of Daily Living
AI	Artificial Intelligence
ANAM	Automated Neuropsychological Assessment Metrics
AUC	Area Under the ROC (Receiver Operating Characteristics) Curve
BHA	Brain Health Assessment
CA	Conventional Assessment
CAAB	Clinical Assessment using Activity Behavior
C-ABC	Computerized Assessment Battery for Cognition
CAMCI	Computer Assessment of Mild Cognitive Impairment
CANS-MCI	Computer-Administered Neuropsychological Screen for Mild Cognitive Impairment
CANTAB	Cambridge Neuropsychological Test Automated Battery
CAVIRE	Cognitive Assessment by Virtual Reality
CCC	Concordance Correlation Coefficients
CCS	Computerized Cognitive Screening
CDT	Clock Drawing Test
CI	Cognitively Impaired
CoCoSc	Computerized Cognitive Screen
CN	Cognitively Normal/Healthy Adult
CST	Computer Self-Test
CT scan	Computed Tomography scan
dTMT	Digital Trail Making Test
eCDT	Electronic Clock Drawing Test
eMoCA	Electronic Montreal Cognitive Assessment
ePDT	Electronic Pentagon Drawing Test
eTMT	Electronic Trail Making Test
FCD	Functional Cognitive Disorder
GPCOG	General Practitioner Assessment of Cognition
HIV	Human Immunodeficiency Viruses
HK-VMT	Hong Kong–Vigilance and Memory Test
IADL	Instrumental Activity of daily living
LABIS	Lawton and Brody IADL scale
MCI	Mild Cognitive Impairment
MRI	Magnetic Resonance Imaging
MIS	Memory Impairment Screen
MMSE	Mini-Mental State Examination
Mini-cog	Mini-Cognitive
MoCA	Montreal Cognitive Assessment
MoCA-K	Korean version of Montreal Cognitive Assessment
MoCA-BJ	Montreal Cognitive Assessment–Beijing version
mSTS-MCI	Mobile Screening Test System for Screening Mild Cognitive Impairment
NNCT	NAIHA Neuro Cognitive Test
NHATS	National Health and Aging Trends Study
PC-based	Personal Computer-based
PD	Parkinson Disease
PDT	Pentagon Drawing Test
PET scan	Positron Emission Tomography
r	Pearson Correlation
r_1_	Bivariate Correlation Coefficients
Saturn	Self-Administered Tasks Uncovering Risk of Neurodegeneration
Sens	Sensitivity
SLE	Systemic Lupus Erythematosus
Spec	Specificity
TIA	Transient Ischemic Attack
TMT	Trail Making Test
UPSA-B	University of California, San Diego Performance-Based Skills Assessment Brief
WAIS-IV	Wechsler Adult Intelligence Scale | Fourth Edition
WTMT	Walking Trail Making Test
VPC	Web-based Visual-Paired Comparison
VRFCAT	Virtual Reality Functional Capacity Assessment Tool
