# Supplementary material for: The Potential of Automated Assessment of Cognitive Function Using Non-Neuroimaging Data: A Systematic Review
Source: J Clin Med. 2024 Nov 22;13(23):7068. doi: 10.3390/jcm13237068 (PMC11642063; doi:10.3390/jcm13237068)
Supplement: Supplementary file 1 [file jcm-13-07068-s001.zip › jcm-3277337-supplementary.pdf]

Performance evaluation of automated cognitive assessment tools based on the 5 categories; Game-based, Digitized version of some conventional tools, Original computerized tests and batteries, Virtual reality/ Wearable sensors/ Smart home technologies, and Artificial intelligence- based (AI-based) techniques

Table S1a: Game-based assessment tools

Name / Biomarker	Country	Participants	Domain assessed	Task	Feature reported	Device used	Reference	Performance measure
Touch panel-type screening test	JP	14 CN, 8 MSA-P, and 25 MSA-C	CD	Flipping cards, and arranging pictures	Accuracy, time taken to perform task	Touch-panel computer	[53]	NA
e-Cube games	US	80 CN	Motor and CD	Shape matching and maze	Correctness and completion time	Computer	[51]	r ≥ 0.09, correlation between each of the 6 adaptive games and 3 subsets of the WAIS-IV
EVO monitor	US	24 CN, 33 MS patients with CI, and 67 MS patients without CI	CD	Perceptual discrimination, and visuomotor tracking	Information processing speed, sustained attention, and motoric quickness	Tablet	[55]	r = 0.5, correlation with SDMT (a subset of the Brief International Cognitive Assessment for Multiple Sclerosis)
MahjongBrain	CN/US	5 participants	CD	Orientation, delayed recall, and drawing	Score and time of completion	Tablet	[54]	NA
Panoramix games	ES	8 CN, 5 AD, and 3 MCI	CD	Playing a sequence of cubes	Number of errors, total playing, and average response time	Computer and touch devices.	[48]	Accu = 1.0
NNCT	ES	70 CN, 44 MCI, and 33 AD	CD	7 cognitive areas in the form of a game	Test score	Tablet	[56]	r = 0.52, correlation with MMSE

AD (Alzheimer Disease), CD (Cognitive Domain), CI (Cognitive Impairment), CN (Control/Healthy adults), MCI (Mild Cognitive Impairment), MS (Multiple Sclerosis), MSA-C (Multiple System Atrophy with predominant cerebellar ataxia), MSA-P (Multiple System Atrophy with predominant parkinsonism), NNCT (NAIHA Neuro Cognitive Test), and WAIS-IV (Wechsler Adult Intelligence Scale | Fourth Edition)

Table S1b: Digitized version of some conventional tools

Name / Biomarker	Country	Participants	Domain assessed	Task	Feature reported	Device used	Reference	Performance measure
mPDT	KR	328 * volunteers and 101 PD patients	CD	Pentagon drawing	Number of angles drawn, and Tremor	Tablet and AI algorithm (U-Net)	[59]	Average values for the features reported: Sens = 0.96, Spec = 0.95, Accu = 0.95, Prec = 0.94
MMSE (App)	BE	15 participants with multimorbidity and impaired functionality	CD	Conventional MMSE with little modification	Speech recognition technique for scoring	m-Health android application	[36]	r = 0.9, correlation with MMSE

eMoCA	US	42 persons with memory complaint	CD	7 domains of MoCA form	Correlation with conventional method	Mobile app (eMoCA) on tablet with stylus	[57]	ccc = 0.84, the score between MoCA and eMoCA. RMSD = 2.27
MoCA-CC	CN	83 CN and 93 MCI	CD	Same as MoCA-BJ	Completion time	Computerized version of MoCA-BJ	[64]	r = 0.93, AUC= 0.97, Sens = 0.958, Spec = 0.871
dTMT	DE	53 CN	CD	Trail making	Completion time and derived scores	12 inches touch screen tablet	[60]	r_1 = 0.87 - 0.89
dTMT for prediction	US	28 CN, 6 MCI, 7 PD, 3 PD/MCI, and 10 with other neurological conditions	CD	Trail making	Time to completion, and number of errors	Tablet and AI algorithm	[61]	r = 0.98, correlation between predicted TMT scores and clinical digital test scores
dTMT & WTMT	CN	7 CN, 7 MCI, and 7 PD	CD	Trail making	Spatial and temporal parameters for human gaits.	Tablets with inertial sensors and pen	[124]	NA
Conventional & digital TMT	FR	68 CN	CD	Trail making part A & B	Time taken for the trail-making task	Tablet, and laptop	[125]	NA
TMT for serious exergame X-Torp (TMTX−Torp)	FR	21 AD, 27 MCI, and 27 CN)	CD		Time to complete the test	Desktop PC	[126]	r ≥ 0.48, correlation between TMTX−Torp and TMT-A & B (completion time)
dCDT	DE	20 AD, 30 MCI, and 20 CN	CD	Clock drawing	Time of stylus in the air	Tablet and stylus pen	[63]	Sens (CA) = 0.63, Spec (CA) = 0.83, Sens (AA) = 0.81, Spec (AA) = 0.72
dCDT	CN	22,567 participants (90 studies)	CD	Clock drawing		A digital pen and a tablet	[35]	Sens (CA) = 0.63, Spec (CA) = 0.77, Sens (AA) = 0.86, Spec (AA) = 0.92
Automated Psychometric test	KR	50 CN	CD	Line tracing test	Line tracing test	e-Pen, & a smart paper printed with location information	[127]	ccc ≥ 0.87, Accu ≥ 0.96

AD (Alzheimer Disease), ccc (Concordance Correlation Coefficients), CD (Cognitive Domain), CI (Cognitive Impairment), CN (Control/Healthy adults), MoCA-BJ (Montreal Cognitive Assessment - Beijing version), MoCA-CC (Computerized tool for the Chinese version of the MoCA), MCI (Mild Cognitive Impairment), PD (Parkinson Disease), r1 (Bivariate Correlation Coefficient), RMSD (Root Mean Squared Difference), and * refers to right-handed participants,

Table S1c: Original computerized tests and batteries

Name / Biomarker	Country	Participants	Domain assessed	Task	Feature reported	Device used	Reference	Performance measure
Cognivue	US	401 participants (at risk of cognitive decline or dementia due to age)	CD	Word, letter and shape discrimination	Visuomotor, perception and memory	Computer	[128]	PPA = 82%, NPA = 98%

CANS-MCI	US	310 elderly participants	CD	Stroop test, and clock test	Executive function, language fluency, and memory	Computer	[129]	r = 0.44 to 0.64, correlation with domain-specific conventional neuropsychological test
BACH computerized screening tool	US	40 CI and 32 CN	CD	Verbal & non-verbal memory test	Response accuracy, & response time	Tablet	[103]	Differentiating CI from CN AUC = 0.78, Sens = 0.80, Spec = 0.72
The Placing Test (Computerized version)	IT	2 groups of 20 CN, 10 MCI, and 9 AD	Episodic memory	Recall association between images, objects, & shapes	Number of items correctly placed	Computer	[73]	r = 0.7,7 correlation between conventional and computerized
Web-based application	US	155 CN	CD	Orientation, face-name hobby recall etc.	Assessment score	Computer	[97]	*r* = 0.77, correlation between pencil-and-paper and web-based measures of self-rated health
Saturn	US	23 Dementia and 37 CN	CD	Orientation, word recall, math etc.	Reading speed and correctly selected answers	Tablet	[98]	r = 0.90, correlation between Saturn and MoCA
C-ABC	JP	422 dementia, 145 MCI, and 134 CN	CD	Touching a moving target, time orientation etc.	Score based on task performed	Touchscreen device	[33]	Sens = 0.77, Spec = 0.71, (average values for distinguishing MCI from CN), r = 0.753, correlation with MMSE score
CANS-MCI	UK	20 CN and 15 MCI	CD	Picture naming & recognition, & word-picture matching	Scores from all tasks	Touchscreen Computer	[91]	AUC = 0.867, Sens = 0.89, Spec = 0.73
Computerized Touch-panel Screening Tests	JP	41 MCI, 124 AD, and 75 CN	CD	4 games (flipping cards, finding mistakes, arranging pictures and beating devils)	Time-taken, and number of errors	Computerized touch-panel	[106]	Sens = 0.769, Spec = 0.77, (for distinguishing CN and MCI using the flipping game at specified cutoff)
MindStreams	IL	454 patients with transient ischemic attack (TIA) or stroke	Neurocognitive decline	Memory, executive function, attention, and motor skills	Cognitive score derived from accuracy and reaction time.	Computer	[130]	r = 0.6 correlation with MoCA
MindStreams	IL	170 participants	CD	Time and place, orientation, and reality testing	Accuracy and reaction time	Computer	[101]	r = 0.77 correlation with MMSE
MindStreams	IL	55 schizophrenia and 63 CN	CD	Stroop test, verbal function and memory	Accuracy, inter-tap interval, and response time	Computer	[131]	0.28 ≤ r ≤ 0.57, and 0.46 ≤ r ≤ 0.59, correlation on raw scores and standardized cognitive scores respectively with CANTAB
Minnemera	SE	81 CN	CD	Attention, executive and function	Timing, pauses, and lifting from the touchscreen	Touch screen device	[27]	0.34 ≤ r ≤ 0.67
CoCoSc	CN	59 CI and 101 CN	CD	Orientation to place test and word list learning	Memory, executive functions, orientation	Touch screen computer	[92]	AUC = 0.78, Sens = 0.78, Spec = 0.69, r = 0.71 correlation with MoCA

CogState	US	37 AD, 7 FTD, 5 DLB, 16, MCI and 22 CN	CD	Detection, identification, & trail test making test	Task performance accuracy and reaction time	Computer	[132]	NA
CST	US	84 CI, 27 MCI, and 104 CN	CD	Clock drawing test, Naming of 15 animals, and recall of current date	Visual-spatial functions, memory, attention, and processing speed	Internet-based	[66]	Sens = 0.99, Spec = 0.95, AUC = 0.99
CCS	IE	20 CN and 40 mild-moderate dementia but only 34 completed the CCS task	CD	Matching pairs of symbols, remember a code from a number/letter sequence, and matching objects shown in different orientations	Concentration, memory and visuospatial function	Tablet	[67]	Sens = 0.94, Spec = 0.60, AUC = 0.94, r = 0.78 correlation with MoCA
mSTS-MCI	KR	103 CN and 74 MCI	CD	Memory, attention, executive function	Reaction time and score	Tablet or computer	[68]	AUC = 0.985, r = 0.773, correlation with MoCA-K, Sens = 0.99, Spec = 0.93, for total scores of mSTS-MCI for detecting MCI
BrainCheck	US	35 CN, 22 MCI, and 42 Dementia	Severity in CI	Trail making, and matching symbols to digits	Memory, executive function, Visual attention	Mobile devices	[102]	Sens ≥ 0.83, Spec ≥ 0.77, AUC ≥ 0.79, Minimum reported among the 3 different groups
MARC	JP	23 AD, 17 MCI, and 24 CN	CD	Object recognition, time orientation, & memory task	Memory and visuospatial function	Touch-panel device	[108]	AUC ≥ 0.866, Minimum reported among the 3 different groups
Sentence-based computerized test	GR	93 MCI and 40 CN	Sentence deficit	Sentence construction and comprehension	Time limit	Computer	[133]	0.259 ≤ r ≤ 0.379, correlation between MMSE and correct answers in the proposed task
VPC	US	44 CN and 11 MCI	CD	Image recall and familiarization	Preference score, eye movement score	Web-based VPC on computer	[134]	r = 0.47, correlation with MoCA
Brain on Track (BoT)	PT	26 MCI, 23 Dementia and 49 CN – Test A 18 MCI, 21 Dementia and 39 CN – Test B	CD	Word categories, Attention, puzzle, and sequences	Correct answers within the time limit	Web-based self-administered test	[109]	AUC = 0.741 (Test – B), AUC = 0.753 (Test – A), **predictive accuracy to distinguish between patients and CN
Computerized real-life activity task (shopping task)	BE	17 hospitalized and 13 non-hospitalized schizophrenia patients and 30 CN	Everyday life functioning	Computerized shopping task	Total time to complete task, number of correct items, number of corrected errors	Computer	[135]	NA
Portuguese PennCNB	PT	152 CN	Neurocognitive domains	14 standard cognitive tasks	Accuracy and speed	Computer	[136]	NA
MITSI-L	US	64 CN and 34 MCI	CD	Word pair recall/learning	Correctness	Touch screen computer	[110]	Sens = 0.853, Spec = 0.844, AUC = 0.93

CogEvo	JP	209 participants	CD	Shape, orientation, and flashlight	Scored based on correct answer and response time	Tablet or a touch screen computer	[100]	0.81 ≤ r ≤ 0.83, correlation with MMSE
CogEvo	JP	40 AD, 38 MCI, and 88 CN	CD	5 tasks (orientation, Visual search, just fit and others)	Speed and accuracy	Touch screen computer	[107]	r = 0.616, correlation with MMSE AUC = 0.83
HK-VMT	CN	97 MCI and 509 CN	CD	Attention test, & delayed matching test	Accuracy based on score and response time	Touch screen computer	[69]	AUC = 0.793, Sens = 0.861, Spec = 0.753
RWLRT	US	249 CN	CD	word recall task	Speech accuracy	Tablet	[137]	0.54 ≤ r ≤ 0.59, correlation with CVLT
SIMBAC	US	10 CN and 10 CI	CD	PhoneTask, Pillbox Task, ATM Task	Correct performance of task	Apple Logic Pro	[99]	r = 0.85 with MMSE, r = 0.76 Lawson IADL, and r = 0.45 with KATZ ADL
BHA Cuban version	CU	53 CN, 46 MCI, and 47 Dementia	CD	Line orientation, animal fluency etc.	Correct score	Tablet-based cognitive battery	[105]	AUC = 0.95, Sens = 0.91, Spec = 0.85. In discriminating between CN and impaired groups (MCI + dementia)
ANAM	CA	300 SLE patients	CD	Tapping right hand, and spatial processing	Mean reaction time and percentage correct scores	ANAM and AI algorithm	[104]	Sens = 0.90, Spec = 0.78, Accu = 0.79, (result from the best model, model 6)
CANTAB	ES	77 aMCI and 85 CN	CD	Automated visual episodic memory tests	PRM, DMS, and PAL	Computerized tool	[11]	Sens = 0.72, Spec = 0.83, Accu = 0.80
CANTAB-PAL	FI	19 AD, 17 aMCI, and 22 CN	CD	CERAD word list learning and PAL task	Word list learning delayed recall, and PAL errors adjusted	Touch screen computer	[12]	AUC = 0.803

AD (Alzheimer Disease), aMCI (amnestic Mild Cognitive Impairment), CD (Cognitive Domain), CI (Cognitive Impairment), CN (Control/Healthy adults), DLB (Dementia with Lewy body), FTD (Frontotemporal Dementia), MARC (mild cognitive impairment (MCI) assessment tool for rapid screening using a computer), MCI (Mild Cognitive Impairment), SLE (Systemic Lupus Erythematosus)

Table S1d: Virtual reality/ Wearable sensors/ Smart home technologies

Name / Biomarker	Country	Participants	Domain assessed	Task	Feature reported	Device used	Reference	Performance measure
CAVE (Cave Automated Virtual Environment)	IT	NA	CD	IADL	Patient's kinematics	ML and VR	[138]	NA
Virtual ADL+ House	SG	8 doctors and 5 healthy older adults	IADLs	IADLs tasks like cleaning designed in the game activities	Time spent to finish each session, the accuracy, and types of errors made	Online VR home environment	[50]	The purpose was to evaluate usability, and positive response was received after

CAAB	US	7 CN, 6 RCD, and 5 ECD	CD and mobility	Cook, eat, and personal hygiene	Behavioral sensor data	Quantitative data from sensors and AI algorithms	[70]	r = 0.72, CAAB-predicted and the clinician provided CS
Smart home technology for automated cognitive assessment	US	16 with Dementia, 51 MCI and 196 CN	CD	Household chores, and medication management	Ability to perform or complete an activity	Smart home sensor data and AI algorithm	[111]	AUC = 0.8, r = 0.54 correlation between observed score and predicted score
CAMCI	US	296 CN and 228 MCI	CI	VR shopping & computerized version of pen-paper test	Attention, working memory, and executive abilities	Touchscreen tablet computer and VR technology	[13]	Sens = 0.86, Spec = 0.94
CAVIRE	SG	125 CN (n =50, 65 to 84 years and n = 75, 34 to 64 years), 50 CI (65 to 84 years)	CD	Virtual tasks such as domestic chores, shopping, and social interactions	Score was based on proportion of tasks performed correctly etc.	Head mounted device for virtual task	[139]	NA
VRFCAT	US	62 schizophrenia patients	Function	IADLs tasks like using the transport, and account management	Adjusted total time, number of errors and number of forced progressions	Computer	[9]	r = 0.005, correlation with baseline UPSA-B,<br>r = 0.010, correlation with PSP Global score
Ecological assessment using serious game-based tool	CH	18 AD patients and 20 CN	CD	6 daily virtual tasks like shopping, and cooking	Time to achieve the task and percentage of completion	Virtual environment using touchscreen computer	[10]	NA
Digital biomarkers	US	80 NCF and 41 CF	CD	Walking	Gait performance	Wearable sensors	[121]	AUC = 0.87
Digital biomarkers	KR	30 CN and 30 MCI	CD	Balanced tasks in 4 different standing postures.	Static balance metrics	Waist-mounted inertial sensor with ML algorithm	[140]	Accu = 0.758 for detecting MCI

AD (Alzheimer Disease), CD (Cognitive Domain), CI (Cognitive Impairment), CN (Control/Healthy adults), CS (Cognitive Score), ECD (Experiencing Cognitive Difficulty), MCI (Mild Cognitive Impairment), ML (Machine Learning), NCF (Non- Cognitive frailty), PSP (Personal and Social Performance scale), RCD (at Risk of Cognitive Difficulty), and UPSA-B (University of California, San Diego Performance-Based Skills Assessment Brief)

Table S1e: Artificial intelligence - based (AI-based) techniques

Name / Biomarker	Country	Participants	Domain assessed	Task	Feature reported	AI technique used	Reference	Performance measure
Automated screening and scoring of CDT images	DE	1315 clock images from the neuro-psychiatric clinic (591 CN and 724 Dementia)	CD	Clock drawing	CDT scoring and screening	DenseNet-121 and Local Linear Embedding (LLE)	[82]	Accu ≥ 0.97 (accuracy for scoring and screening)

Memtrax	CN	101 CN, 158 MCI	CD	Online episodic memory test	Digital voice and screen-based behaviors	ML algorithms (Naïve Bayes performed best)	[84]	AUC > 0.7
Automated evaluation of CDT	JP	40,131 CDT images from NHATS database	CD	CDT	Cognitive decline	Deep Neural Network	[74]	Accu ~ 0.90 (executive dysfunction), Accu = 0.77 (Dementia)
Using CNN to predict CI	KR	747 CDT and 980 RCFT - copy figures	CD	Copying the "Rey complex figure" and clock drawing	Visual constructional function, clock semantics and inhibitory function	Convolutional neural network	[75]	Accu = 0.71
Explainable DNN for detecting CI	TH	651 CN and 267 MCI	CD	CDT, cube-copying, and TMT	Classification based on the drawing	Deep neural network (VGG 16)	[81]	Accu = 0.81, F1-score = 0.65, AUC = 0.84
AI-assisted tool for detecting CI	JP	23 CI, and 55 CN	CD	Daily activity	Daily activity data (with power monitoring)	Machine Learning (Factorial Hidden Markov Models)	[85]	Accu = 0.82, Sens = 0.48, Spec = 0.96
Multi-feature automated Speech analysis	US	28 MCI and 42 CN	CD	Audio recording	Count-based and time-based	Automatic speech recognition (ASR) and Support Vector Machine	[78]	AUC = 0.77
Digital speech assessment	CA	14 MCI/AD, 18 with high MoCA score and 18 with low MoCA score	CD	Digital recording	Word finding difficulty, sentiments, and lexical richness	Natural language processing (Winter light Labs speech analysis platform)	[79]	r = 0.47, correlation of value of information content scores and MoCA scores
Automated speech analysis (ASA)	CA	30 speech samples (10 each from AD, MCI, and CN with 1 MCI participant providing 2 samples)	Language impairment	Speech recording	Word-finding difficulty and incoherence	Natural language processing (NLP) and Automated speech analysis (ASA)	[80]	ICC ≥ 0.91
Speech-based	IT	48 CN and 48 aMCI	CD	Speech of subjects describing 3 tasks was recorded and transcribed	Spoken texts, lexical, and syntactic features	NLP techniques	[15]	NA
Speech analysis to detect CI	US/ES	87 CN, 63 MCI, and 24 AD	CD	Tasks on animal fluency, alternating fluency etc.	Speech analysis	Ensemble of Logistic Regression, Supported Vector Machines, K-Nearest Neighbors, Random Forest and Gradient Boosting	[76]	AUC = 0.93, Accu = 0.884, Sens = 0.875, Spec = 0.892 CN with impaired (MCI + dementia)
Eye movement analysis to detect CI	US	30 CN, 10 MCI and 20 AD	CD	Gaze positions	Patterns of fixations, saccade orientation etc.	Support Vector Machine	[115]	Accu = 0.87, Sens = 0.97, Spec = 0.77
Digital voice biomarker	US	92 CN and 114 CI	CD	Audio recordings using ML approach	Lexical semantic and acoustic scores	Natural Language Processing	[77]	AUC = 0.77 and 0.80 for Acoustic score and Lexical semantic

								respectively. These were higher than Boston Naming Test (BNT)'s AUC of 0.66 in detecting MCI
CNN to discriminate MCI	KR	354 RCFT images (copy and recall conditions) from 103 CN and 74 a-MCI	CD	RCFT image copy and recall	Prediction of MCI based on the RCFT-copy and RCFT-recall images	Convolutional Neural Network	[83]	Accu ≥ 0.846, AUC ≥ 0.851 For RCFT recall and copy. Accu = 0.818, AUC = 0.848 For MoCA-K
Digital psychological features	SG	17 aMCI	CD	106 digital physiological features	Physiological features	Linear Mixed-Effect Regression (LMER)	[86]	r = 0.94, correlation between NTB (Neuropsychological test batteries) and digital features.
DL-based CI prediction using multi-center NPTs	KR	14,926 formal neuropsychological assessments (3217 CN, 6002 MCI and 5707 AD)	CD	Multi-center NPT data	46-features of formal neuropsychological data	Artificial Neural Network	[116]	Accu, Sens and Spec > 0.94
Integrated Cognitive Assessment (ICA)	UK	95 CN, 80 MCI, and 55 mild AD	CD	Visual categorization task	Reaction time and image categorization accuracy	Logistic Regression	[119]	r = 0.58, correlation with MMSE AUC of 0.81 and 0.88 for detecting MCI and mild AD respectively
Memory Guard (MG)	CN	183 CN and 150 MCI	CD	Orientation, and attention	Option scores, and time scores	Combination of AdaBoost with the Naive Bayes	[114]	Accu = 0.9375, Sens = 0.9167, Spec = 0.9545, AUC = 0.923
Dementia classification using clinical and cognitive measures	AR, CO, CL, MX, and PE	904 AD, 282 FTD and 606 CN	CD	Clinical, demographic and cognitive data	Social cognition, and executive functioning	Random forest	[112]	Accu = 0.91 Classifying AD and FTD
ML-based VR (VR-Supermarket)	TW	6 MCI or early AD and 6 CN	Executive function	Virtual shopping task	Task performance (number of items over-buy or less-buy)	Logistic regression and Support vector machine	[113]	Accu = 1.00
CognoSpeak	UK	15 AD, 15 MCI, 15 FMD and 15 CN	CD	Verbal response to virtual clinician	Word vector, lexical, and acoustic features	Automatic Speech Recognition (ASR)	[120]	Sens = 0.867, Spec = 0.767, Result for 2-way classification
ML-based approach of predicting CI	KR	3424 CN	CD	Sociodemographic, health, and functional status	Age, health satisfaction, and limited daily activity	Gradient Boosting Machine	[118]	Sens = 0.967, Spec = 0.825, AUC = 0.921
MyCog	US	67 CN and 19 CI	CD	Matching visual images	Executive function, episodic memory	Random Forest and Artificial Neural Network	[34]	Prec = 0.803, Recall = 0.758, Accu = 0.902, F1-score = 0.742, Spec = 0.951
Physical function indication with clinical prediction modelling	CN	5393 participants	CD	Handgrip Strength, Self-Rated Squat Ability etc.	Behavioral factors, mental status, lower and upper limb function	Logistic regression	[117]	0.719 ≤ AUC ≤ 0.732 for the 3 predictive models

Panoramix games	ES	8 CN, 5 AD, and 3 MCI	CD	Playing a sequence of cubes	Number of errors, total playing, and average response time	Machine learning (support Vector Machine (SVM) and Classification and Regression Trees (CART)	[48]	Accu = 1.0
mPDT	KR	328 * volunteers and 101 PD patients	CD	Pentagon drawing	Number of angles drawn, and Tremor	U-Net, Deep5 and DeepLock	[59]	Average values for the features reported: Sens = 0.96, Spec = 0.95, Accu = 0.95, Prec = 0.94
dTMT for prediction	US	28 CN, 6 MCI, 7 PD, 3 PD/MCI, and 10 with other neurological conditions	CD	Trail making	Time to completion, and number of errors	Support vector machine	[61]	r = 0.98, between predicted TMT scores and clinical digital test scores
ANAM	CA	300 SLE patients	CD	Tapping right hand, and spatial processing	Mean reaction time and percentage correct scores	Classification and Regression Tree (CART) analysis decision tree	[104]	Sens = 0.90, Spec = 0.78, Accu = 0.79, (result from the best model, model 6)
CAVE (Cave Automated Virtual Environment)	IT	NA	CD	IADL	Patient's kinematics	Machine learning	[138]	NA
CAAB	US	7 CN, 6 RCD, and 5 ECD	CD and mobility	Cook, eat, and personal hygiene	Behavioral sensor data	Support vector machine	[70]	r = 0.72, correlation with clinician provided CS
Smart home technology for automated cognitive assessment	US	16 with Dementia, 51 MCI and 196 CN	CD	Household chores, and medication management	Ability to perform or complete an activity	Support vector machine	[111]	AUC = 0.8, r = 0.54, correlation between observed score and predicted score
Digital biomarkers	KR	30 CN and 30 MCI	CD	Balanced tasks in 4 different standing postures.	Static balance metrics	Support vector machine	[140]	Accu = 0.758 for detecting MCI

aMCI (amnestic Mild Cognitive Impairment), BACH (Brief Assessment of Cognitive Health), CD (Cognitive Domain), CNN (Convolutional Neural Network), CRAE (Central Retinal Artery Equivalent), CRVE (Central Retinal Vein Equivalent), CS (Cognitive Score), DLB (Dementia with Lewy body), DMS (Delayed Matching to Sample), DNN (Deep Neural Network), ECD (Experiencing Cognitive Difficulty), FMD (Functional Memory Disorder), FTD (Frontotemporal Dementia), ICC (Intra-class Correlations), MARC (mild cognitive impairment (MCI) assessment tool for rapid screening using a computer), ML (Machine Learning), MoCA-CC (Computerized tool for the Chinese version of the MoCA), MS (Multiple Sclerosis), MSA-C (Multiple System Atrophy with predominant cerebellar ataxia), MSA-P (Multiple System Atrophy with predominant parkinsonism), NAIHA (Natural and Artificial Intelligence Health Assistant), NCF (Non- Cognitive frailty), NLP (Natural Language Processing), PAL (Paired Associated Learning), PANSS (Positive and Negative Syndrome Scale, PRM (Pattern recognition memory), PSP (Personal and Social Performance scale), r1 refers to the Bivariate Correlation Coefficient, RCD (at Risk of Cognitive Difficulty), RCFT (Rey–Osterrieth Complex Figure Test), RMSD (Root Mean Squared Difference), SCD (Subjective cognitive decline), * refers to right-handed participants, +2-way classification, ++3-way classification, and +++4-way classification.

Argentina	Belgium	Canada	Switzerland	Chile	China	Colombia	Cuba	Germany	Spain	Finland	France	Greece	Ireland	Israel	Italy	Japan	Korea	México	Perú	Portugal	Sweden	Singapore	Thailand	Taiwan	United Kingdom	USA
AR	BE	CA	CH	CL	CN	CO	CU	DE	ES	FI	FR	GR	IE	IL	IT	JP	KR	MX	PE	PT	SE	SG	TH	TW	UK	US

Disclaimer/Publisher's Note: The statements, opinions and data contained in all publications are solely those of the individual author(s) and contributor(s) and not of MDPI and/or the editor(s). MDPI and/or the editor(s) disclaim responsibility for any injury to people or property resulting from any ideas, methods, instructions or products referred to in the content.
